# Supplementary figures and images for: Early identification of preterm neonates at birth with a Tablet App for the Simplified Gestational Age Score (T-SGAS) when ultrasound gestational age dating is unavailable: A validation study
Source: PLoS One. 2020 Aug 31;15(8):e0238315. doi: 10.1371/journal.pone.0238315 (PMC7458295; doi:10.1371/journal.pone.0238315)

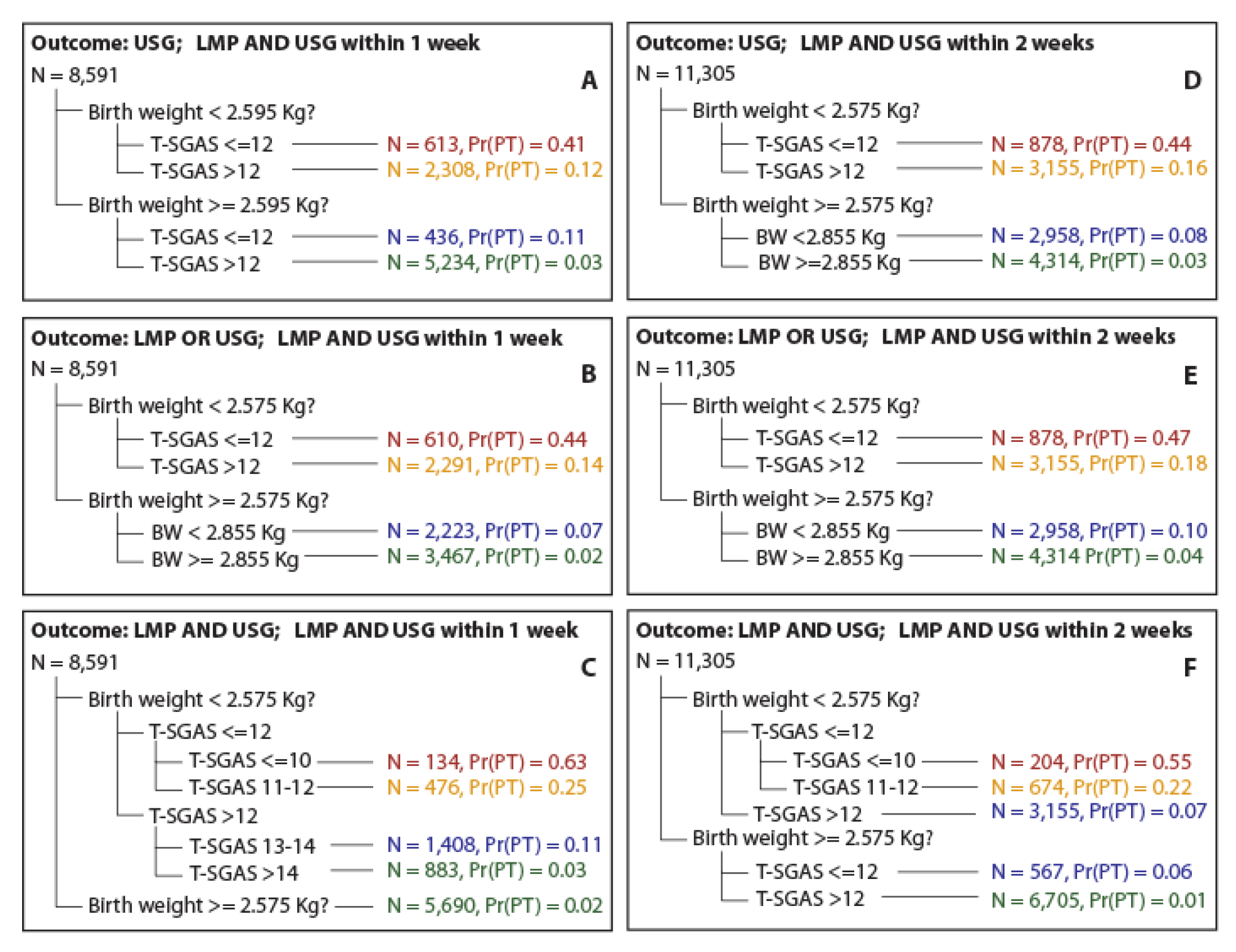

Supplement: S1 Fig — (TIF) [file pone.0238315.s001.tif]

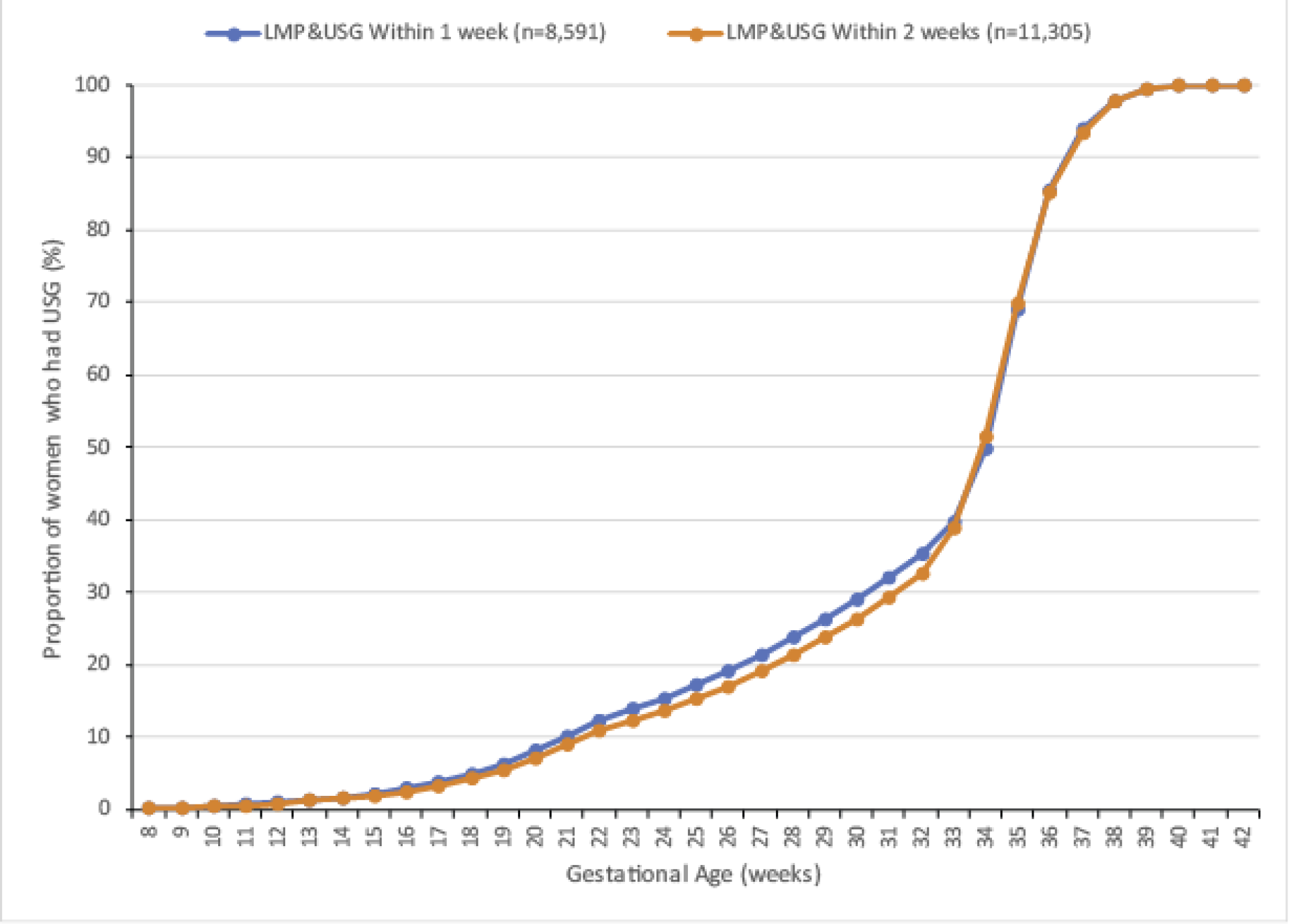

Supplement: S2 Fig — (TIF) [file pone.0238315.s002.tif]

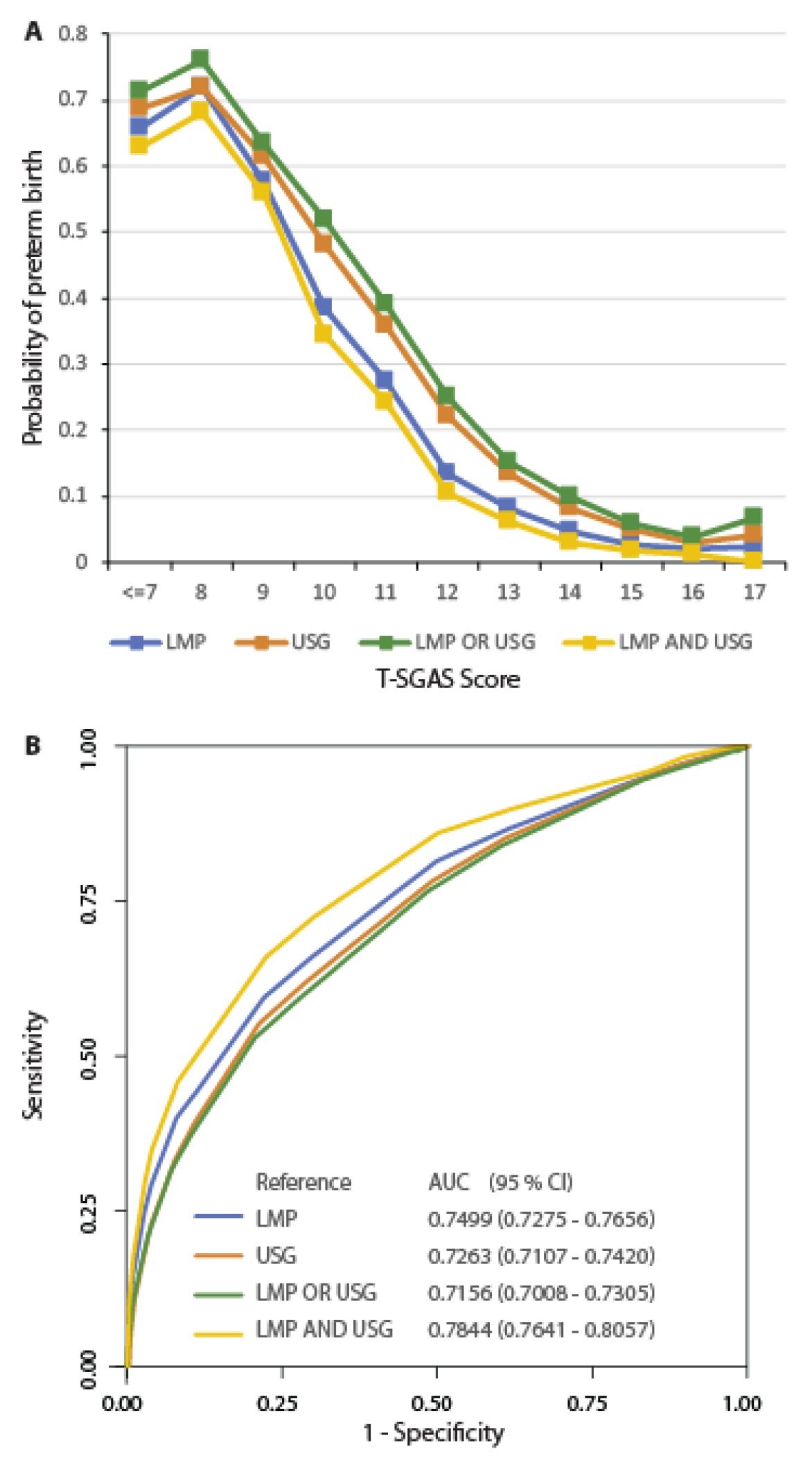

Supplement: S3 Fig — (TIF) [file pone.0238315.s003.tif]

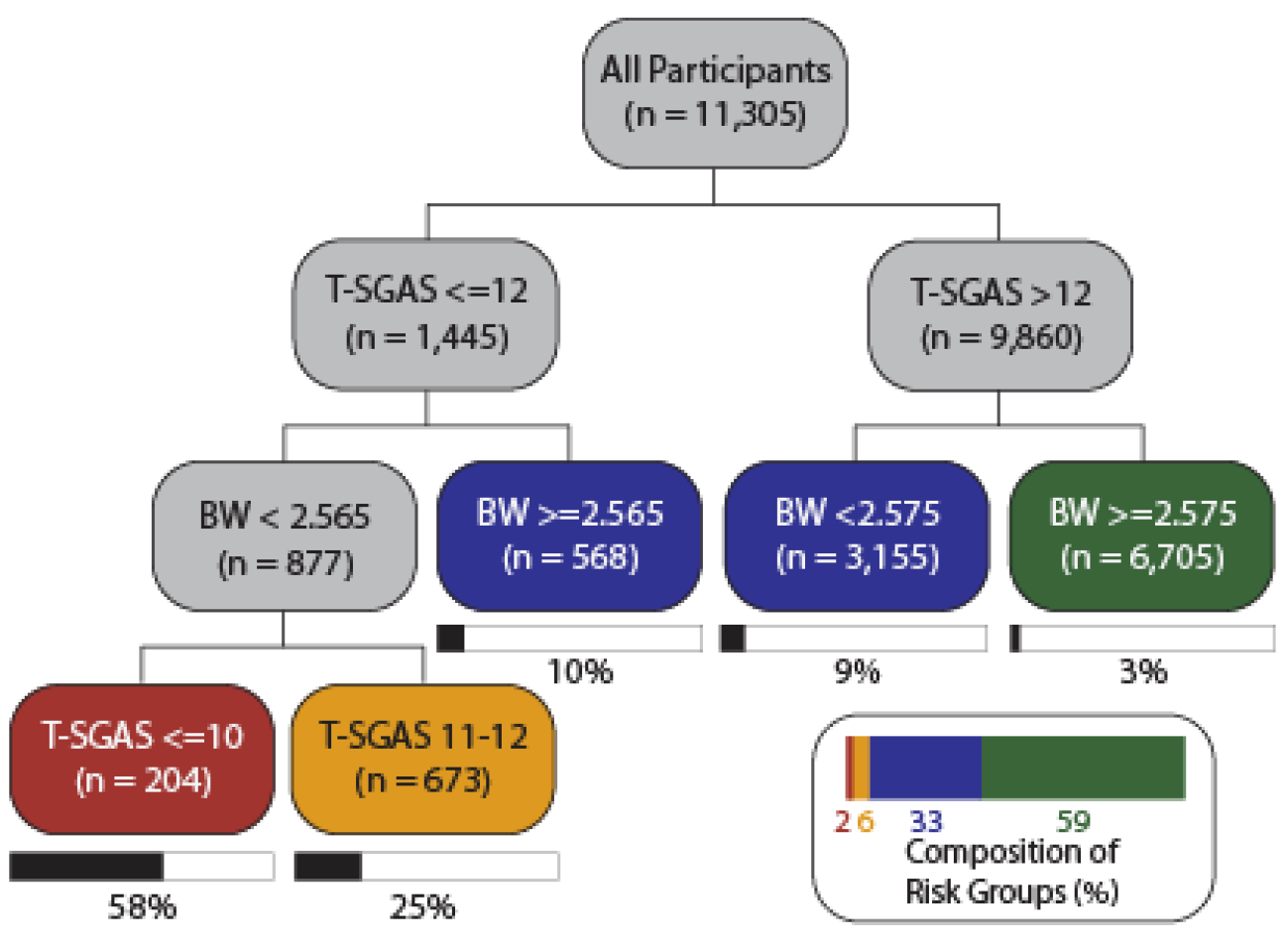

Supplement: S4 Fig — (TIF) [file pone.0238315.s004.tif]
