# Supplementary material for: Early identification of preterm neonates at birth with a Tablet App for the Simplified Gestational Age Score (T-SGAS) when ultrasound gestational age dating is unavailable: A validation study
Source: PLoS One. 2020 Aug 31;15(8):e0238315. doi: 10.1371/journal.pone.0238315 (PMC7458295; doi:10.1371/journal.pone.0238315)
Supplement: S1 Table — (DOCX) [file pone.0238315.s005.docx]

**Table S1: Equivalence between included and excluded participants.**

| **Variable** | **Included Participants**  **(n = 11,305)** | **Excluded Participants**  **(n = 4,615)** |
| --- | --- | --- |
| Enrolling facility [n (%)]  -Daga  -Bhandara  -Wardha | 6,753 (58.14)  3,448 (30.50)  1,284 (11.36) | 2,897 (62.77)  1,193 (25.85)  525 (11.38) |
| Maternal age [mean (SD)] y | 24.30 (3.31) | 24.14 (3.31) |
| Nulliparous [n (%)] | 5,459 (48.29) | 2,040 (44.82) |
| Parity [median (IQR)] | 1 (1) | 1 (1) |
| Maternal education [n (%)]  -None  -Primary  -Secondary  -Higher | 40 (0.35)  727 (6.43)  5,850 (51.75)  4,688 (41.47) | 31 (0.68)  597 (13.11)  2,380 (52.27)  1,545 (33.93) |
| Mode of delivery  -Vaginal  -Caesarean section  -Vaginal assisted | 5,914 (52.31)  5,083 (44.96)  308 (2.72) | 2,570 (56.45)  1,810 (39.75)  173 (3.80) |
| GA at first USG [median (IQR)] w | 34 (3) | 34 (2) |
| Birth weight [mean (SD)] Kg | 2.75 (0.40) | 2.61 (0.44) |
| Foot length [mean (SD)] cm | 9.06 (11.57) | 9.16 (12.17) |
| Female gender [n (%)] | 5,394 (47.71) | 2,329 (50.47) |
| T-SGAS score [mean (SD)]  -First Assessor  -Second Assessor | 14.08 (1.53)  14.06 (1.53) | 13.73 (1.88)  13.75 (1.84) |
