# Supplementary material for: Early identification of preterm neonates at birth with a Tablet App for the Simplified Gestational Age Score (T-SGAS) when ultrasound gestational age dating is unavailable: A validation study
Source: PLoS One. 2020 Aug 31;15(8):e0238315. doi: 10.1371/journal.pone.0238315 (PMC7458295; doi:10.1371/journal.pone.0238315)
Supplement: S2 Table — (DOCX) [file pone.0238315.s006.docx]

**Table S2: Screening accuracy of T-SGAS to identify preterm births when estimates of GA by LMP and USG were within 2 weeks of each other (n = 11,305).**

| **Parameter** | **Reference Standard** | | | |
| --- | --- | --- | --- | --- |
|  | **LMP** | **USG** | **LMP OR USG** | **LMP AND USG** |
| *Assessor 1: Estimates of GA by LMP & USG within 2 weeks* | | | | |
| Prevalence | 7.04 (6.58 - 7.53) | 10.89 (10.32 - 11.48) | 12.60 (11.99 - 13.22) | 5.33 (4.93 - 5.76) |
| Sensitivity | 42.71 (39.25 - 46.23) | 37.61 (34.90 - 40.39) | 35.60 (33.11 - 38.15) | 49.09 (45.03 - 53.16) |
| Specificity | 89.49 (88.88 - 90.07) | 90.25 (89.66 - 90.82) | 90.51 (89.91 - 91.08) | 89.26 (88.66 - 89.84) |
| PPV | 23.53 (21.36 - 25.80) | 32.04 (29.64 - 34.52) | 35.09 (32.62 - 37.61) | 20.48 (18.43 - 22.66) |
| NPV | 95.38 (94.94 - 95.78) | 92.21 (91.66 - 92.73) | 90.70 (90.11 - 91.27) | 96.89 (96.52 - 97.22) |
| LR + | 4.06 (3.68 - 4.48) | 3.86 (3.51 - 4.24) | 3.75 (3.42 - 4.11) | 4.57 (4.15 - 5.04) |
| LR - | 0.64 (0.60 - 0.68) | 0.69 (0.66 - 0.72) | 0.71 (0.68 - 0.74) | 0.57 (0.53 - 0.62) |
| *Assessor 2: Estimates of GA by LMP & USG within 2 weeks* | | | | |
| Prevalence | 7.04 (6.58 - 7.53) | 10.89 (10.32 - 11.48) | 12.60 (11.99 - 13.22) | 5.33 (4.93 - 5.76) |
| Sensitivity | 43.09 (39.62 - 46.61) | 36.72 (34.02 - 39.48) | 35.11 (32.63 - 37.66) | 48.92 (44.86 - 52.99) |
| Specificity | 89.39 (88.79 - 89.97) | 90.01 (89.41 - 90.59) | 90.30 (89.70 - 90.88) | 89.13 (88.53 - 89.72) |
| PPV | 23.53 (21.37 - 25.79) | 31.00 (28.63 - 33.45) | 34.29 (31.86 - 36.79) | 20.23 (18.20 - 22.39) |
| NPV | 95.40 (94.97 - 95.80) | 92.09 (91.54 - 92.61) | 90.62 (90.02 - 91.19) | 96.87 (96.51 - 97.21) |
| LR + | 4.06 (3.69 - 4.48) | 3.68 (3.35 - 4.04) | 3.62 (3.30 - 3.97) | 4.50 (4.08 - 4.97) |
| LR - | 0.64 (0.60 - 0.68) | 0.70 (0.67 - 0.73) | 0.72 (0.69 - 0.75) | 0.57 (0.53 - 0.62) |

PPV, positive predictive value; NPV, negative predictive value; LR +, likelihood ratio of a positive test; LR -, likelihood ratio of a negative test
