# Supplementary material for: Early identification of preterm neonates at birth with a Tablet App for the Simplified Gestational Age Score (T-SGAS) when ultrasound gestational age dating is unavailable: A validation study
Source: PLoS One. 2020 Aug 31;15(8):e0238315. doi: 10.1371/journal.pone.0238315 (PMC7458295; doi:10.1371/journal.pone.0238315)
Supplement: S3 Table — (DOCX) [file pone.0238315.s007.docx]

**Table S3: Stratified Mantel-Haenszel analyses to investigate the potential influence of enrolling institutions on estimates of T-SGAS accuracy.**

| **Assessor** | **LMP & USG within** | **Reference standard** | **Crude estimates** | | **M-H estimates** | | **Heterogeneity*** | |
| --- | --- | --- | --- | --- | --- | --- | --- | --- |
|  |  |  | **Sensitivity** | **Specificity** | **Sensitivity** | **Specificity** | **Chi-sq_het_** | **P_het_** |
| 1 | 2 weeks | LMP | 42.71 | 89.49 | 43.00 | 88.50 | 8.45 | 0.0146 |
| 1 | 2 weeks | USG | 37.61 | 90.25 | 38.31 | 89.69 | 1.73 | 0.4207 |
| 1 | 2 weeks | LMP OR USG | 35.60 | 90.51 | 36.31 | 89.85 | 3.76 | 0.1529 |
| 1 | 2 weeks | LMP AND USG | 49.09 | 89.26 | 49.06 | 88.34 | 1.43 | 0.4880 |
| 1 | 1 week | LMP | 43.27 | 90.07 | 43.50 | 89.11 | 2.06 | 0.3574 |
| 1 | 1 week | USG | 42.45 | 90.51 | 42.85 | 89.84 | 1.12 | 0.5705 |
| 1 | 1 week | LMP OR USG | 40.05 | 90.70 | 40.57 | 89.94 | 0.64 | 0.7264 |
| 1 | 1 week | LMP AND USG | 47.51 | 89.89 | 47.54 | 89.01 | 0.10 | 0.9491 |
| 2 | 2 weeks | LMP | 43.09 | 89.39 | 43.01 | 89.34 | 3.02 | 0.2205 |
| 2 | 2 weeks | USG | 36.72 | 90.01 | 36.77 | 89.93 | 9.85 | 0.0072 |
| 2 | 2 weeks | LMP OR USG | 35.11 | 90.30 | 35.14 | 90.25 | 7.38 | 0.0250 |
| 2 | 2 weeks | LMP AND USG | 48.92 | 89.13 | 48.73 | 89.03 | 3.78 | 0.1508 |
| 2 | 1 week | LMP | 44.63 | 90.22 | 44.56 | 90.09 | 1.37 | 0.5036 |
| 2 | 1 week | USG | 41.47 | 90.47 | 41.42 | 90.37 | 5.10 | 0.0781 |
| 2 | 1 week | LMP OR USG | 39.43 | 90.69 | 39.39 | 90.60 | 3.00 | 0.2226 |
| 2 | 1 week | LMP AND USG | 48.76 | 90.01 | 48.63 | 89.86 | 2.06 | 0.3568 |

Considering the 16 tests of heterogeneity conducted here, the Bonferroni corrected type I error rate below which the p-values were considered significant was 0.0031. At this cut-off none of the p-values showed significant heterogeneity.
