# Supplementary material for: Early identification of preterm neonates at birth with a Tablet App for the Simplified Gestational Age Score (T-SGAS) when ultrasound gestational age dating is unavailable: A validation study
Source: PLoS One. 2020 Aug 31;15(8):e0238315. doi: 10.1371/journal.pone.0238315 (PMC7458295; doi:10.1371/journal.pone.0238315)
Supplement: S4 Table — (DOCX) [file pone.0238315.s008.docx]

**Table S4: Stratified Mantel-Haenszel analyses to investigate the potential influence of the gender of newborn on estimates of T-SGAS accuracy.**

| **Assessor** | **LMP & USG within** | **Reference standard** | **Crude estimates** | | **M-H estimates** | | **Heterogeneity*** | |
| --- | --- | --- | --- | --- | --- | --- | --- | --- |
|  |  |  | **Sensitivity** | **Specificity** | **Sensitivity** | **Specificity** | **Chi-sq_het_** | **P_het_** |
| 1 | 2 weeks | LMP | 42.71 | 89.49 | 42.72 | 89.48 | 0.29 | 0.5905 |
| 1 | 2 weeks | USG | 37.61 | 90.25 | 37.66 | 90.26 | 0.13 | 0.7153 |
| 1 | 2 weeks | LMP OR USG | 35.60 | 90.51 | 35.62 | 90.51 | 0.01 | 0.9403 |
| 1 | 2 weeks | LMP AND USG | 49.09 | 89.26 | 49.12 | 89.27 | 1.60 | 0.2057 |
| 1 | 1 week | LMP | 43.27 | 90.07 | 43.28 | 90.07 | 0.00 | 0.9715 |
| 1 | 1 week | USG | 42.45 | 90.51 | 42.48 | 90.53 | 0.03 | 0.8572 |
| 1 | 1 week | LMP OR USG | 40.05 | 90.70 | 40.04 | 90.71 | 0.59 | 0.4419 |
| 1 | 1 week | LMP AND USG | 47.51 | 89.89 | 47.54 | 89.89 | 0.62 | 0.4297 |
| 2 | 2 weeks | LMP | 43.09 | 89.39 | 43.11 | 89.38 | 0.03 | 0.8711 |
| 2 | 2 weeks | USG | 36.72 | 90.01 | 36.78 | 90.03 | 0.01 | 0.9148 |
| 2 | 2 weeks | LMP OR USG | 35.11 | 90.30 | 35.15 | 90.30 | 0.03 | 0.8517 |
| 2 | 2 weeks | LMP AND USG | 48.92 | 89.13 | 48.96 | 89.15 | 0.39 | 0.5326 |
| 2 | 1 week | LMP | 44.63 | 90.22 | 44.66 | 90.21 | 0.18 | 0.6702 |
| 2 | 1 week | USG | 41.47 | 90.47 | 41.52 | 90.50 | 0.64 | 0.4252 |
| 2 | 1 week | LMP OR USG | 39.43 | 90.69 | 39.46 | 90.70 | 1.29 | 0.2562 |
| 2 | 1 week | LMP AND USG | 48.76 | 90.01 | 48.80 | 90.03 | 0.00 | 0.9467 |

Considering the 16 tests of heterogeneity conducted here, the Bonferroni corrected type I error rate below which the p-values were considered significant was 0.0031. At this cut-off none of the p-values showed significant heterogeneity.
