# Supplementary material for: Early identification of preterm neonates at birth with a Tablet App for the Simplified Gestational Age Score (T-SGAS) when ultrasound gestational age dating is unavailable: A validation study
Source: PLoS One. 2020 Aug 31;15(8):e0238315. doi: 10.1371/journal.pone.0238315 (PMC7458295; doi:10.1371/journal.pone.0238315)
Supplement: S5 Table — (DOCX) [file pone.0238315.s009.docx]

**Table S5: Stratified Mantel-Haenszel analyses to investigate the potential influence of maternal age quartiles on estimates of T-SGAS accuracy.**

| **Assessor** | **LMP & USG within** | **Reference standard** | **Crude estimates** | | **M-H estimates** | | **Heterogeneity*** | |
| --- | --- | --- | --- | --- | --- | --- | --- | --- |
|  |  |  | **Sensitivity** | **Specificity** | **Sensitivity** | **Specificity** | **Chi-sq_het_** | **P_het_** |
| 1 | 2 weeks | LMP | 42.71 | 89.49 | 42.78 | 89.46 | 2.52 | 0.4719 |
| 1 | 2 weeks | USG | 37.61 | 90.25 | 37.67 | 90.22 | 1.01 | 0.7977 |
| 1 | 2 weeks | LMP OR USG | 35.60 | 90.51 | 35.68 | 90.48 | 1.10 | 0.7779 |
| 1 | 2 weeks | LMP AND USG | 49.09 | 89.26 | 49.07 | 89.23 | 1.11 | 0.7740 |
| 1 | 1 week | LMP | 43.27 | 90.07 | 43.35 | 90.06 | 2.43 | 0.4878 |
| 1 | 1 week | USG | 42.45 | 90.51 | 42.51 | 90.50 | 1.83 | 0.6093 |
| 1 | 1 week | LMP OR USG | 40.05 | 90.70 | 40.11 | 90.70 | 1.46 | 0.6919 |
| 1 | 1 week | LMP AND USG | 47.51 | 89.89 | 47.51 | 89.89 | 3.19 | 0.3627 |
| 2 | 2 weeks | LMP | 43.09 | 89.39 | 43.12 | 89.39 | 2.34 | 0.5053 |
| 2 | 2 weeks | USG | 36.72 | 90.01 | 36.73 | 90.00 | 0.09 | 0.9925 |
| 2 | 2 weeks | LMP OR USG | 35.11 | 90.30 | 35.13 | 90.30 | 0.26 | 0.9678 |
| 2 | 2 weeks | LMP AND USG | 48.92 | 89.13 | 48.90 | 89.13 | 1.50 | 0.6827 |
| 2 | 1 week | LMP | 44.63 | 90.22 | 44.68 | 90.22 | 2.14 | 0.5438 |
| 2 | 1 week | USG | 41.47 | 90.47 | 41.47 | 90.47 | 0.41 | 0.9382 |
| 2 | 1 week | LMP OR USG | 39.43 | 90.69 | 39.45 | 90.69 | 0.14 | 0.9873 |
| 2 | 1 week | LMP AND USG | 48.76 | 90.01 | 48.74 | 90.01 | 2.64 | 0.4503 |

Considering the 16 tests of heterogeneity conducted here, the Bonferroni corrected type I error rate below which the p-values were considered significant was 0.0031. At this cut-off none of the p-values showed significant heterogeneity.
