# Supplementary material for: Early identification of preterm neonates at birth with a Tablet App for the Simplified Gestational Age Score (T-SGAS) when ultrasound gestational age dating is unavailable: A validation study
Source: PLoS One. 2020 Aug 31;15(8):e0238315. doi: 10.1371/journal.pone.0238315 (PMC7458295; doi:10.1371/journal.pone.0238315)
Supplement: S6 Table — a. Results of latent class analyses (analysis subset: LMP and USG estimates of GA within one week of each other, n = 8,591). b. Results of latent class analyses when estimates of GA by LMP and USG were within 2 weeks of each other (n = 11,305). (DOCX) [file pone.0238315.s010.docx]

**Table S6a: Results of latent class analyses(Analysis subset: LMP and USG estimates of GA within one week of each other, n = 8,591)**

| Parameter | Assessor 1 | Assessor 2 |
| --- | --- | --- |
| Prevalence (95% CI) | 7.74 (6.93 – 8.56) | 7.32 (6.56 – 8.09) |
| Sensitivity (95% CI) |  |  |
| T-SGAS | 47.57 (43.09 – 52.05) | 48.84 (44.35 – 53.33) |
| LMP | 78.33 (72.67 – 83.99) | 83.37 (77.91 – 88.83) |
| USG | 92.34 (88.02 – 96.66) | 91.68 (87.39 – 95.98) |
| Specificity (95% CI) |  |  |
| T-SGAS | 90.76 (90.11 – 91.41) | 90.73 (90.09 – 91.38) |
| LMP | 99.17 (98.83 – 99.50) | 99.22 (98.89 – 99.54) |
| USG | 98.81 (98.29 -99.32) | 98.34 (97.96 – 98.82) |

**Table S6b: Results of latent class analyses when estimates of GA by LMP and USG were within 2 weeks of each other (n = 11,305).**

| **Parameter** | **Assessor 1** | **Assessor 2** |
| --- | --- | --- |
| Prevalence (95% CI) | 8.51 (7.63 – 9.39) | 8.38 (7.50 – 9.26) |
| Sensitivity (95% CI) |  |  |
| T-SGAS | 49.40 (45.33 – 53.46) | 49.23 (45.16 – 53.29) |
| LMP | 68.89 (63.48 – 74.30) | 71.01 (65.48 – 76.54) |
| USG | 90.24 (90.04 – 94.50) | 88.93 (84.56 – 93.31) |
| Specificity (95% CI) |  |  |
| T-SGAS | 90.63 (90.04 – 91.21) | 90.42 (89.83 – 91.02) |
| LMP | 98.71 (98.37 – 99.06) | 98.81 (98.46 – 99.16) |
| USG | 96.50 (95.85 – 97.14) | 96.25 (95.62 – 96.88) |

| Reference standard  used to define  Preterm | Integrated Discrimination Improvement, p | Net Reclassification Index, p |
| --- | --- | --- |
| *Assessor 1* | | |
| LMP alone | 0.0672, <0.0001 | 0.4609, <0.0001 |
| USG alone | 0.0644, <0.0001 | 0.5204, <0.0001 |
| LMP OR USG | 0.0610, <0.0001 | 0.4710, <0.0001 |
| LMP AND USG | 0.0758, <0.0001 | 0.5185, <0.0001 |
| *Assessor 2* | | |
| LMP alone | 0.0709, <0.0001 | 0.4956, <0.0001 |
| USG alone | 0.0613, <0.0001 | 0.4731, <0.0001 |
| LMP OR USG | 0.0601, <0.0001 | 0.4465, <0.0001 |
| LMP AND USG | 0.0774, <0.0001 | 0.5333, <0.0001 |
