# Supplementary material for: Early identification of preterm neonates at birth with a Tablet App for the Simplified Gestational Age Score (T-SGAS) when ultrasound gestational age dating is unavailable: A validation study
Source: PLoS One. 2020 Aug 31;15(8):e0238315. doi: 10.1371/journal.pone.0238315 (PMC7458295; doi:10.1371/journal.pone.0238315)
Supplement: S7 Table — a. Incremental value of T-SGAS over birth weight (analysis subset: LMP and USG estimates of GA within one week of each other, n = 8,591). b. Incremental value of T-SGAS over birth weight when estimates of GA by LMP and USG were within 2 weeks of each other (n = 11,305). (DOCX) [file pone.0238315.s011.docx]

| Reference standard  used to define  Preterm | Integrated Discrimination Improvement, p | Net Reclassification Index, p |
| --- | --- | --- |
| *Assessor 1* | | |
| LMP alone | 0.0672, <0.0001 | 0.4609, <0.0001 |
| USG alone | 0.0644, <0.0001 | 0.5204, <0.0001 |
| LMP OR USG | 0.0610, <0.0001 | 0.4710, <0.0001 |
| LMP AND USG | 0.0758, <0.0001 | 0.5185, <0.0001 |
| *Assessor 2* | | |
| LMP alone | 0.0709, <0.0001 | 0.4956, <0.0001 |
| USG alone | 0.0613, <0.0001 | 0.4731, <0.0001 |
| LMP OR USG | 0.0601, <0.0001 | 0.4465, <0.0001 |
| LMP AND USG | 0.0774, <0.0001 | 0.5333, <0.0001 |

**Table S7a: Incremental value of T-SGAS over birth weight(Analysis subset: LMP and USG estimates of GA within one week of each other, n = 8,591)**

**Table S7b: Incremental value of T-SGAS over birth weight when estimates of GA by LMP and USG were within 2 weeks of each other (n = 11,305).**

| **Reference standard**  **used to define**  **Preterm** | **Incremental value** | |
| --- | --- | --- |
|  | **IDI, p** | **NRI, p** |
| *Assessor 1* | | |
| LMP alone | 0.0606, <0.0001 | 0.4565, <0.0001 |
| USG alone | 0.0471, <0.0001 | 0.3945, <0.0001 |
| LMP OR USG | 0.0474, <0.0001 | 0.4146, <0.0001 |
| LMP AND USG | 0.0695, <0.0001 | 0.4997, <0.0001 |
| *Assessor 2* | | |
| LMP alone | 0.0571, <0.0001 | 0.4304, <0.0001 |
| USG alone | 0.0448, <0.0001 | 0.3912, <0.0001 |
| LMP OR USG | 0.0453, <0.0001 | 0.3719, <0.0001 |
| LMP AND USG | 0.0648, <0.0001 | 0.4523, <0.0001 |
