# Supplementary material for: Early identification of preterm neonates at birth with a Tablet App for the Simplified Gestational Age Score (T-SGAS) when ultrasound gestational age dating is unavailable: A validation study
Source: PLoS One. 2020 Aug 31;15(8):e0238315. doi: 10.1371/journal.pone.0238315 (PMC7458295; doi:10.1371/journal.pone.0238315)
Supplement: S8 Table — (DOCX) [file pone.0238315.s012.docx]

**Table S8: Combinations of ANM Assessors who evaluated the live births.**

| First Assessor Code | Second Assessor Code | | | | | | Total |
| --- | --- | --- | --- | --- | --- | --- | --- |
|  | 13 | 14 | 15 | 16 | 17 | 18 |  |
| 1 | 5 | 1 | 0 | 385 | 442 | 0 | 833 |
| 2 | 0 | 0 | 0 | 449 | 491 | 0 | 940 |
| 3 | 0 | 3 | 0 | 475 | 376 | 0 | 854 |
| 4 | 507 | 0 | 0 | 0 | 0 | 0 | 507 |
| 5 | 0 | 236 | 413 | 1 | 0 | 513 | 1,163 |
| 6 | 0 | 80 | 443 | 0 | 1 | 453 | 977 |
| 7 | 0 | 129 | 560 | 0 | 0 | 571 | 1,260 |
| 8 | 0 | 0 | 669 | 0 | 0 | 577 | 1,246 |
| 9 | 0 | 0 | 486 | 0 | 0 | 282 | 768 |
| 10 | 0 | 0 | 596 | 0 | 0 | 562 | 1,158 |
| 11 | 0 | 0 | 0 | 402 | 425 | 0 | 827 |
| 12 | 771 | 0 | 1 | 0 | 0 | 0 | 772 |
| Total | 1,283 | 449 | 3,168 | 1,712 | 1,735 | 2,958 | 11,305 |
