# Supplementary material for: Early identification of preterm neonates at birth with a Tablet App for the Simplified Gestational Age Score (T-SGAS) when ultrasound gestational age dating is unavailable: A validation study
Source: PLoS One. 2020 Aug 31;15(8):e0238315. doi: 10.1371/journal.pone.0238315 (PMC7458295; doi:10.1371/journal.pone.0238315)
Supplement: S9 Table — (DOCX) [file pone.0238315.s013.docx]

**Table S9: Agreement between assessor pairs when estimates of GA by LMP and USG were within 2 weeks of each other (n = 11,305).**

| **Description** | **Agreement** |
| --- | --- |
| Agreement between assessors |  |
| Complete agreement [n (%)] | 6,765 (59.8) |
| 1 point [n (%)] | 3,188 (28.2) |
| 2 points [n (%)] | 959 (8.5) |
| 3 points [n (%)] | 261 (2.3) |
| 4+ points [n (%)] | 132 (1.2) |
| Fleiss’s kappa (95% CI) | 0.77 (0.75 – 0.78) |
| Agreement between assessments |  |
| Complete agreement [n (%)] | 9,638 (85.3) |
| Fleiss’s kappa (95% CI) | 0.69 (0.67 – 0.70) |
