# Supplementary material for: Early identification of preterm neonates at birth with a Tablet App for the Simplified Gestational Age Score (T-SGAS) when ultrasound gestational age dating is unavailable: A validation study
Source: PLoS One. 2020 Aug 31;15(8):e0238315. doi: 10.1371/journal.pone.0238315 (PMC7458295; doi:10.1371/journal.pone.0238315)
Supplement: Attachment — Submitted filename: T-SGAS_ Response to Reviewer_July11, 2020.docx [file pone.0238315.s014.docx]

Please include the following items when submitting your revised manuscript:

- A rebuttal letter that responds to each point raised by the academic editor and reviewer(s). This letter should be uploaded as separate file and labeled 'Response to Reviewers'.
- A marked-up copy of your manuscript that highlights changes made to the original version. This file should be uploaded as separate file and labeled 'Revised Manuscript with Track Changes'.
- An unmarked version of your revised paper without tracked changes. This file should be uploaded as separate file and labeled 'Manuscript'.

**RESPONSE TO REVIEWER**

Additional Editor Comments (if provided):

The submission titled "Early identification of preterm neonates at birth with a Tablet App for the Simplified Gestational Age Score (T-SGAS) when ultrasound gestational age dating is unavailable: A validation study" by Archana Patel et al is an attempt to come up with a screening tool to estimate gestational age of neonates in a resource poor setting so that special attention may be given to infants born <37 weeks to prevent morbidity and mortality. Authors report on use of a android tablet app by two trained midwife's in 4 community hospitals in India to assess gestational age with an aim to designate each infant as either <37 weeks or >37 weeks. This tool becomes highly relevant in countries like India where majority of deliveries are conducted by nurse midwife's and an assessment of GA helps screen neonates who need special attention and referral to an appropriate tertiary care centre. This is a well-designed study and a well-written manuscript and has the potential for widespread use in the third world countries. I would like authors to make several major and minor changes to their submission before it can be considered suitable for publication.

**Response:** We are thankful to you for careful review and understanding the relevance of this paper in India.

Major concerns:
1. T-SGAS a pictorial App that consisted of photographs to score four items: newborn’s posture (score 0 to +4), skin (score - 134 1 to +5), breast (score -1 to +4) and genitals (score -1 to +4), seems very interesting compared to other established tools like Dubowitz or Modified Ballard. It will be very helpful to our readers if authors could add a figure to the manuscript to depict the images used to do this said scoring. In its current form this manuscript does not provide a visual impression of the assessment being made to our readers.

**Response:**

1. *We have added the visual impression of the T-SGAS in the manuscript’s method section as figure 1. In our earlier referenced paper - “Patel A, Kurhe K, Prakash A, Bhargav S, Parepalli S, Fogleman E et al. Development and Validation of a Tablet-based Simplified Gestational Age Score (T-SGAS) for Early Identification of Preterm Neonates at Birth when Ultrasound Gestational Age Dating is Not Available: A Global Network for Maternal and Child Health Research Protocol. Available at:* [*http://preprints.jmir.org/preprint/11913*](http://preprints.jmir.org/preprint/11913) *DOI: 10.2196/preprints.11913.” we have described in detail how this simplified score was adapted with pictures on a tablet.*

2. Was the GA assessment also done by established norms like Dubowitz or Ballard's methods by a trained professional? If yes, then how these assessments varied compared to the app? If not then why not? Kindly explain it in the discussion part of the manuscript.

**Response:**

*The Simplified Gestational Age Score development on the tablet underwent the following two steps –*

*Step - 1) We evaluated the 12-item New Ballard Score (NBS), 22-item Dubowitz score (DWS) and 11-item Meharban Singh (MS) and assessed the correlation of the total of these 3 scores and each item in these 3 scores with the Best obstetrics Estimate (BOE) (LMP and USG as gold standard). (Ref. Patel A, Lothe A, Belekar N, Thakur H. Development and initial validation of a simplified gestational age score in low birth weight newborns in India. Indian Journal of Child Health 2017;5(1): 15-19.) Through this exercise, we developed a 4-item simplified gestational age score (SGAS) for use in low birth weight newborns in India. SGAS showed promising results for accurate estimation of GA in LBW newborn and ease of use by nursing staff in the community.*

*Step – 2) We adapted the 4-item simplified gestational age score (SGAS) to a pictorial tablet app. This method was described in our referenced paper - “Patel A, Kurhe K, Prakash A, Bhargav S, Parepalli S, Fogleman E et al. Development and Validation of a Tablet-based Simplified Gestational Age Score (T-SGAS) for Early Identification of Preterm Neonates at Birth when Ultrasound Gestational Age Dating is Not Available: A Global Network for Maternal and Child Health Research Protocol. Available at:* [*http://preprints.jmir.org/preprint/11913*](http://preprints.jmir.org/preprint/11913) *DOI: 10.2196/preprints.11913.”*

*In the current study the ANMs were trained to use only the T-SGAs. The aim of this study was to assess if ANMs who otherwise have minimal training in assessment of gestational age are able to use the T-SGAS reliably to assess GA of newborns at birth. It was validated against the gold standard of GA at birth assessed using prenatal USG, which is also the gold standard for validating any newborn gestatational age assessment methods like NBS or any other scoring method. So validation against NBS or DWS was not considered for two reasons – 1) Any neonatal GA assessment score is a less accurate method for assessment of GA than prenatal USG, 2) Any additional training provided to ANMs on NBA or DWS would bias the evaluation skills of ANMs.*

*This has now been explained in the discussion section.*

3. Discussion needs to elaborate on how this tool can be used by ANM's and in what way it can be helpful to these designated infants <37 weeks?

**Response:**

*We have explained in the methods and discussion section that the ANM has to simply select the picture on the Tablet App that matches the what they observed in the newborn baby. The App then auto-calculated the score which categorizes the newborn as preterm < 37 weeks. The T-SGAS had high reliability and high enough likelihood ratios to warrant its routine use by ANMs to refer preterm newborns to higher facilties.*

4. Table 3 and 4 though helpful to a trained professional with good knowledge of biostatistics, does not help our average reader of PLOSone so should be either deleted or published as supplementary tables on the web.

**Response:**

*We have now moved table 3 and 4 to supplement. It is now S6a and S7a respectively*

Minor concerns:
1. Remove repetitive statements in introduction, methods and results to reduce the size of manuscript.

**Response:**

*We have done as suggested.*

2. Elaborate on educational status on ANM's in your methods section and why they were chosen to do the measurements, were they randomly selected or had special qualifications?

**Response:**

*The educational level of ANMs is a diploma in midwifery of 2 years provided after high school education. The reason behind selecting, ANM’s for this study was that they provide midwifery service at all rural primary health centers. They were selected on random basis and trained in use of the T-SGAS App*

Journal requirements:

When submitting your revision, we need you to address these additional requirements:

1.    Please ensure that your manuscript meets PLOS ONE's style requirements, including those for file naming. The PLOS ONE style templates can be found at http://www.plosone.org/attachments/PLOSOne_formatting_sample_main_body.pdf and http://www.plosone.org/attachments/PLOSOne_formatting_sample_title_authors_affiliations.pdf

**Response:**

*We have done the needful.*

2. Please provide additional details regarding participant consent. In the Methods section, please ensure that you have specified what type of consent you obtained (for instance, written or verbal) and whether the ethics committee approved this consent procedure. If verbal consent was obtained please state why it was not possible to obtain written consent and how verbal consent was recorded. If your study included minors, state whether you obtained consent from parents or guardians.

**Response:**

*We have now provided the details in the methods section.*

3. PLOS requires an ORCID iD for the corresponding author in Editorial Manager on papers submitted after December 6th, 2016. Please ensure that you have an ORCID iD and that it is validated in Editorial Manager. To do this, go to ‘Update my Information’ (in the upper left-hand corner of the main menu), and click on the Fetch/Validate link next to the ORCID field. This will take you to the ORCID site and allow you to create a new iD or authenticate a pre-existing iD in Editorial Manager. Please see the following video for instructions on linking an ORCID iD to your Editorial Manager account: https://www.youtube.com/watch?v=_xcclfuvtxQ

**Response:**

*ORCID iD-0000-0002-25587421 (Dr. Archana Patel) It has been validated in the editorial manager*.

4. We note that you have indicated that data from this study are available upon request. PLOS only allows data to be available upon request if there are legal or ethical restrictions on sharing data publicly. For information on unacceptable data access restrictions, please see <http://journals.plos.org/plosone/s/data-availability#loc-unacceptable-data-access-restrictions>.

**Response:**

*Indian Council of Medical Research does not allow data sharing unless the protocol for data sharing was previously approved by them, the Institutional Ethics Committee and consented by the participants. If data is requested by the journal for verification of results we will seek permissions from the Institutional Ethics Committee (LMRF-IEC), our Sponsors and the Indian Council of Medical. However, other researchers may submit data access requests to Dr. Prabir Kumar Das, Member Secretary at LMRF-IEC (prabir_das23@rediffmail.com).”*

In your revised cover letter, please address the following prompts:

 a) If there are ethical or legal restrictions on sharing a de-identified data set, please explain them in detail (e.g., data contain potentially identifying or sensitive patient information) and who has imposed them (e.g., an ethics committee). Please also provide contact information for a data access committee, ethics committee, or other institutional body to which data requests may be sent.

**Response:**

*Please see our response above. The contact information for data access our –*

*LMRF-IEC:*

*Dr Prabir Kumar Das*

*Member Secretary*

*Lata Medical Research Foundation,*

*Kinkini Kutir, Vasantnagar, Nagpur – 440022*

*Ph. No. 91-8805023450*

*Email ID- Prabir_das23@rediffmail.com*

b) If there are no restrictions, please upload the minimal anonymized data set necessary to replicate your study findings as either Supporting Information files or to a stable, public repository and provide us with the relevant URLs, DOIs, or accession numbers. Please see http://www.bmj.com/content/340/bmj.c181.long for guidelines on how to de-identify and prepare clinical data for publication. For a list of acceptable repositories, please see http://journals.plos.org/plosone/s/data-availability#loc-recommended-repositories.

**Response:**

*Please see previous response.*

5. Thank you for stating the following in the Competing Interests section:

"The authors declare no competing interests."

We note that one or more of the authors are employed by a commercial company: M&H Research, LLC

1. Please provide an amended Funding Statement declaring this commercial affiliation, as well as a statement regarding the Role of Funders in your study. If the funding organization did not play a role in the study design, data collection and analysis, decision to publish, or preparation of the manuscript and only provided financial support in the form of authors' salaries and/or research materials, please review your statements relating to the author contributions, and ensure you have specifically and accurately indicated the role(s) that these authors had in your study. You can update author roles in the Author Contributions section of the online submission form.

**Response:**

*While Dr. Kulkarni is affiliated to M&H Research, LLC, San Antonio, TX, USA; this work was done under the aegis of Lata Medical Research Foundation, Nagpur, India of which Dr. Kulkarni is the President and provides his services gratis. No funding was received from M&H Research, LLC for work reported here.*

Please also include the following statement within your amended Funding Statement.

*“**The funder provided support to the institute for conduct of the study, but did not have any additional role in the study design, data collection and analysis, decision to publish, or preparation of the manuscript. The specific roles of these authors are articulated in the ‘author contributions’ section.”*

If your commercial affiliation did play a role in your study, please state and explain this role within your updated Funding Statement.

**Response**:

*The commercial affiliation had no role to play in our study.*

2. Please also provide an updated Competing Interests Statement declaring this commercial affiliation along with any other relevant declarations relating to employment, consultancy, patents, products in development, or marketed products, etc.

 Within your Competing Interests Statement, please confirm that this commercial affiliation does not alter your adherence to all PLOS ONE policies on sharing data and materials by including the following statement: "This does not alter our adherence to  PLOS ONE policies on sharing data and materials.” (as detailed online in our guide for authors [http://journals.plos.org/plosone/s/competing-interests](about:blank)) . If this adherence statement is not accurate and  there are restrictions on sharing of data and/or materials, please state these. Please note that we cannot proceed with consideration of your article until this information has been declared.

Please include both an updated Funding Statement and Competing Interests Statement in your cover letter. We will change the online submission form on your behalf.

Please know it is PLOS ONE policy for corresponding authors to declare, on behalf of all authors, all potential competing interests for the purposes of transparency. PLOS defines a competing interest as anything that interferes with, or could reasonably be perceived as interfering with, the full and objective presentation, peer review, editorial decision-making, or publication of research or non-research articles submitted to one of the journals. Competing interests can be financial or non-financial, professional, or personal. Competing interests can arise in relationship to an organization or another person. Please follow this link to our website for more details on competing interests: [http://journals.plos.org/plosone/s/competing-interests](about:blank)

6. We note that you have included the phrase “data not shown” in your manuscript. Unfortunately, this does not meet our data sharing requirements. PLOS does not permit references to inaccessible data. We require that authors provide all relevant data within the paper, Supporting Information files, or in an acceptable, public repository. Please add a citation to support this phrase or upload the data that corresponds with these findings to a stable repository (such as Figshare or Dryad) and provide and URLs, DOIs, or accession numbers that may be used to access these data. Or, if the data are not a core part of the research being presented in your study, we ask that you remove the phrase that refers to these data.

**Response:**

*We have provided an explanation regarding the data sharing requirement. We shall remove the phrase.*

7. Your ethics statement must appear in the Methods section of your manuscript. If your ethics statement is written in any section besides the Methods, please move it to the Methods section and delete it from any other section. Please also ensure that your ethics statement is included in your manuscript, as the ethics section of your online submission will not be published alongside your manuscript.

**Response:**

*This has been updated in the method section.*

8. Please include captions for your Supporting Information files at the end of your manuscript, and update any in-text citations to match accordingly. Please see our Supporting Information guidelines for more information: [http://journals.plos.org/plosone/s/supporting-information](about:blank).

**Response:**

*This has now been provided.*

[Note: HTML markup is below. Please do not edit.]

Reviewers' comments:

Reviewer's Responses to Questions

**Comments to the Author**

1. Is the manuscript technically sound, and do the data support the conclusions?

The manuscript must describe a technically sound piece of scientific research with data that supports the conclusions. Experiments must have been conducted rigorously, with appropriate controls, replication, and sample sizes. The conclusions must be drawn appropriately based on the data presented.

Reviewer #1: Yes

2. Has the statistical analysis been performed appropriately and rigorously?

Reviewer #1: Yes

3. Have the authors made all data underlying the findings in their manuscript fully available?

The [PLOS Data policy](http://www.plosone.org/static/policies.action#sharing) requires authors to make all data underlying the findings described in their manuscript fully available without restriction, with rare exception (please refer to the Data Availability Statement in the manuscript PDF file). The data should be provided as part of the manuscript or its supporting information, or deposited to a public repository. For example, in addition to summary statistics, the data points behind means, medians and variance measures should be available. If there are restrictions on publicly sharing data—e.g. participant privacy or use of data from a third party—those must be specified.

Reviewer #1: Yes

4. Is the manuscript presented in an intelligible fashion and written in standard English?

PLOS ONE does not copyedit accepted manuscripts, so the language in submitted articles must be clear, correct, and unambiguous. Any typographical or grammatical errors should be corrected at revision, so please note any specific errors here.

Reviewer #1: Yes

5. Review Comments to the Author

Please use the space provided to explain your answers to the questions above. You may also include additional comments for the author, including concerns about dual publication, research ethics, or publication ethics. (Please upload your review as an attachment if it exceeds 20,000 characters)

Reviewer #1: This is a cross-sectional study evaluating the accuracy of Tablet based gestational age estimation app in low resource setting. They have identified score <14 as good predictor of identifying preterm birth (<37 weeks) and 9% probability of preterm birth if score>13 and weight <2.5kg.

The app has high Inter & intra-observer variation and Likelihood ratio, though sensitivity is low. The study is well designed and the analysis is robust and potential for being a useful tool in low-resource settings.

Concerns:
1. 37 weeks was used as the cutoff for identifying preterm birth or not. How valid is the tool at other gestational ages - <32 weeks, <35 weeks etc. <37 weeks is important for epidemiological reasons to identify preterm birth. However, infants >35 weeks generally don’t require admission to NICU. This data would be more helpful for clinicians whether the baby needs to be transported to the NICU or not, which in a low resource NICU could be a significant cost.

**Response:**

*This is an interesting suggestion by the Reviewer. However, the suggested analyses are outside the scope of the current manuscript. We do nothave data on how many of the newborn babies needed to admitted to NICU since the study was conducted in primary health care settings. However, we do understand that our tool can have further improved implication if we do additional studies on the lines of the Reviewer’s suggestion. We have now added this as an implication in the Discussion section.*

2. Can you clarify if the ANM assessor’s correlation coefficient was for assessors from all three study sites?

**Response:**

*This is correct.*

3. Likelihood ratios should be included in the abstract.

**Response:**

*This has been updated in the abstract.*
